# Perspectives on Precision Psychiatry Using Antipsychotics in the Management of Bipolar Disorder

**DOI:** 10.3390/brainsci15050430

**Published:** 2025-04-23

**Authors:** Michele Fornaro, Alessandro Miola, Domenico De Berardis, Alessio Squassina, Mirko Manchia, Marco Solmi

**Affiliations:** 1 Department of Neurosciences, Section of Psychiatry, Federico II University of Naples, 80131 Naples, Italy; 2 Department of Neuroscience, University of Padova, 35122 Padua, Italy; alessandro.miola@gmail.com; 3 Department of Psychiatry & Psychology, Mayo Clinic, Rochester, MN 55905, USA; 4 Department of Mental Health, ASL 4, 64100 Teramo, Italy; domenico.deberardis@aslteramo.it; 5 Section of Psychiatry, Department of Medical Sciences and Public Health, University of Cagliari, 09127 Cagliari, Italy; squassina@unica.it (A.S.); mirko.manchia@unica.it (M.M.); 6 Unit of Clinical Psychiatry, University Hospital Agency of Cagliari, 09127 Cagliari, Italy; 7 Department of Pharmacology, Dalhousie University, Halifax, NS B3H 2Y9, Canada; 8 SCIENCES Lab, Department of Psychiatry, University of Ottawa, Ottawa, ON K1N 6N5, Canada; marco.solmi83@gmail.com; 9 Regional Centre for the Treatment of Eating Disorders and On Track: The Champlain First Episode Psychosis Program, Department of Mental Health, The Ottawa Hospital, Ottawa, ON K1H 8L6, Canada; 10 Clinical Epidemiology Program, Ottawa Hospital Research Institute (OHRI), University of Ottawa, Ottawa, ON K1N 6N5, Canada; 11 Department of Child and Adolescent Psychiatry, Charité Universitätsmedizin, 10117 Berlin, Germany

**Keywords:** precision medicine, antipsychotic, bipolar disorder, shared decision-making, psychopharmacology

## Abstract

Background/Objectives: Precision medicine is not just hype. Instead, it represents a high bar for developing more effective, safer, and better-tolerated therapies in medicine, without exception in psychiatry, including bipolar disorder (BD). A burgeoning body of narrative reviews and perspective papers has already appraised the boundaries of precision medicine in BD. Methods: This brief perspective follows a narrative, critical approach focusing explicitly on the antipsychotic management of BD using precision approaches. Results: While most controversies align with those previously appraised in BD’s overall precision medicine approach, specific insights are provided herein. Conclusions: Beyond other implications and the strengthened call for valid diagnostic coding systems, the implementation of shared decision-making tools and pharmacogenomics studies focusing on persons with BD are particularly warranted.

## 1. Introduction

[…“Tonight, I’m launching a new precision medicine initiative to bring us closer to curing diseases like cancer and diabetes. … And to give us all access to the personalized information to keep ourselves and our families healthier”.]—former U.S. President Obama, 2015 State of the Union Address—20 January 2015.

Over a decade has passed since that presidential initiative, which, among other implications, reignited interest in the 2009 U.S. National Institutes of Health (NIH)—promoting research domain criteria (RDoC) project [1] aimed at pursuing precision medicine in psychiatry [2,3].

The RDoC initiative is primarily devoted to research rather than clinical practice [4] and aims to promote validity over reliability. This approach differs from the existing *Diagnostic and Statistical Manual of Mental Disorders* (DSM, various editions) and the *International Classification of Diseases* (ICD, various releases) frameworks for psychiatric diagnoses, including bipolar disorder (BD) [5].

However, as already critically appraised elsewhere, the diagnostic boundaries of current diagnostic systems are just one of many issues hindering precision medicine for BD [6,7,8,9].

This challenge is particularly pressing given that BD is a prevalent, severe mental illness (SMI) [10] characterized by a highly polygenic architecture involving multiple genes with small individual effects alongside various environmental factors that contribute to the cumulative disease risk [11,12]. These elements result in significant clinical heterogeneity and different treatment trajectories [13] across different interventions, including antipsychotic medications, whose prescription rates have steadily increased over the past decades, especially second-, rather than first-generation, antipsychotics for BD [14,15,16].

The present perspective briefly reappraises the boundaries toward precision antipsychotic management of BD, providing a critical overview.

## 2. Current Scenarios: The Pitfalls of Evidence-Based Antipsychotic Management of Bipolar Disorder

Existing treatment guidelines for BD are indispensable resources for the prescribing clinician, and recommendations about antipsychotic drugs are no exception. Yet, they may fail to capture the complexity of real-world clinical practice [17,18], calling for a precision medicine approach [19].

Existing treatment guidelines, among other issues, rely on placebo-controlled randomized trials (RCTs) or their meta-analytic synthesis. Whilst informative, placebo-controlled RCTs only provide evidence on individual treatments, are primarily designed to compare an active intervention versus placebo, and have limited generalizability [20]. Network meta-analyses (NMAs) can overcome the former issue by allowing for indirect comparisons of active interventions [21]. Yet, they must rely on stringent assumptions, including transitivity, meaning that the known potential confounding factors, such as setting, sample characteristics, and treatment modalities, should hold stable across different RCTs, ultimately joining a shared network to allow for a credible synthesis of evidence [22], including antipsychotic management of BD. Regarding the limited generalizability of RCTs, severe presentations of BD, especially those featuring an early age-at-onset of illness, rapid-cycling course with a high risk for suicidality, mixed features and multiple comorbidities (e.g., substance use disorder, attention-deficit hyperactivity disorder, obsessive-compulsive and general medical diseases) [23,24], as well as complex polypharmacy [25], treatment-resistance [26] and strong predominant polarity [27], almost invariably serve as exclusion criteria (or are often overlooked) in RCTs of interventions for BD, including those evaluating antipsychotic medications [28]. This results in a well-known selection bias [29].

Furthermore, the exact proportion of real-world patients with BD who may fail to meet the inclusion criteria of an average RCT is yet to be systematically appraised. Also, in RCTs, treatment adherence is optimized since several incentives facilitate better compliance from the participant, in addition to the selected nature of the samples, which leaves those not interested in treatment out. However, in the real world, roughly one person out of two does not take medications as directed [30]. Thus, our confidence in translating RCT-derived evidence to real-world clinical practice is limited to inferential estimates. In this regard, it is worth noticing that roughly three out of four patients with a primary diagnosis of schizophrenia, a condition for which cornerstone pharmacological treatment involves antipsychotic medications, seen in real-world settings may not be captured by pertinent RCTs acknowledged by treatment guidelines [31].

The lack of representativeness of RCTs to the real world could be a potentially significant obstacle to precision antipsychotic management of BD, too, considering the complexity and heterogeneity of patients with BD [32,33]. The suboptimal efficacy, safety, and tolerability profile of antipsychotic treatments for BD further compounds these challenges, as shown in depth across BD phases in several NMAs, i.e., acute bipolar depression [34,35,36], acute mania [37,38,39], or the maintenance phase of BD [40,41].

Pragmatic trials [42] and systematic reviews could control for moderators and mediators of drug response in BD, including antipsychotics [43]. Nonetheless, they have been proposed to bridge the gap between “ideal world” efficacy trials aimed at maximizing internal validity over external generalizability and large simple trials that maximize external validity at the expense of precision to better reflect real-world clinical practice.

## 3. Additional Boundaries Toward Precision Antipsychotic Management of Bipolar Disorder

While the reader has already been prompted to refer to specific critical reviews about precision medicine in BD, including, but not necessarily limited to, the following sources [6,7,8,9], we briefly recall the essential issues relevant to the antipsychotic management of BD herein.

The development of effective and safer antipsychotics for BD continues to represent a daunting task due to the limited understanding of the putative neurobiological underpinnings [44], especially concerning cellular and animal models of BD overall [45]. Moreover, most efforts toward a better understanding of the neurobiology and search for putative biomarkers of BD focus on lithium [46], aiming at precision pharmacogenomics [47] rather than antipsychotics (as detailed elsewhere [48]) since, in essence, the latter drugs have been considered multidimensional treatments [49] compared to lithium (yet not ubiquitously effective across different presentations of BD) [50]. Among other implications, the lack of specificity of most psychiatric drugs, including antipsychotics, across different SMIs—including BD—is a well-known barrier toward precision medicine, prompting enriched clinical trials with patients assessed for predictive biomarkers such as inflammatory ones and for the use of clinical outcome assessments that align with immune-inflammatory effects on the central nervous system [51]. In particular, BD has a significant genetic loading, contributing to substantial variability in antipsychotic drug response [11]. Therefore, pharmacogenomics remains a crucial avenue [8,52] toward precision antipsychotic management of BD, especially regarding tolerability rather than efficacy considerations, as already acknowledged [7].

Indeed, two sets of genetic data, one based on common variation and the other on cytochrome P450 (CYP) genes, might help inform the management of antipsychotic treatment, especially in BD.

Concerning genomic data, a recent genome-wide association study (GWAS) [53] showed that polygenic risk scores composed of single-nucleotide polymorphisms (SNPs) associated with antipsychotic response in GWAS studies could predict clinical responses to these drugs in a real-world sample of patients with different diagnoses, including BD. However, the predictive accuracy was suboptimal and not yet clinically applicable. Another study focused on antipsychotic tolerability in BD applying pathway analysis to GWAS data from a subsample of the systematic treatment enhancement program for bipolar disorder (STEP-BD) study, Ref. [54] found that molecular pathways related to cell survival events and lipid synthesis were significantly associated with antipsychotic-induced extrapyramidal symptoms. Finally, in an intriguing follow-up analysis of GWAS data [55], Qi et al. (2023) tested whether BD GWAS risk genes were targets of existing drugs or novel compounds that could be repurposed in the clinical treatment of BD. The authors found 58 BD GWAS risk gene targets of antipsychotics, antidepressants, antiepileptics, calcium channel antagonists, as well as anxiolytics and analgesics, either existing clinically approved drugs or drugs that can be repurposed for the treatment of BD in the future. In sum, genomic-informed management of antipsychotic therapy in BD remains in its infancy. Still, it is plausible that predictive accuracy will improve with access to larger datasets and the application of artificial intelligence [AI]-driven follow-up analyses.

However, it is essential to highlight that, to date, none of the genes identified in GWAS studies as being involved in the response or safety of antipsychotic treatment in BD have been incorporated into drug labels approved by the U.S. Food and Drug Administration (FDA). Nevertheless, several genes encoding cytochromes involved in the liver metabolization of antipsychotics are listed in the drug labels of 10 antipsychotic medications (https://www.fda.gov/drugs/science-and-research-drugs/table-pharmacogenomic-biomarkers-drug-labeling, accessed on 21 April 2025). The information provided in these labels includes recommendations based on evidence suggesting that genotype-based information on CYPs metabolizing profiles could help guide treatment decisions. According to PharmGKB (https://www.pharmgkb.org), a pharmacogenetic information repository combining recommendations from drug regulatory agencies and those from the international consortia engaged with the curation of pharmacogenetic guidelines (Clinical Pharmacogenetics Implementation Consortium (CPIC)) and the Dutch Pharmacogenetics Working Group (DPWG)), five antipsychotic gene combinations currently hold the highest possible level for clinical annotations (“1A”; see Table 1).

Level 1A is only reached when variant-drug combinations have variant-specific prescribing guidance available in a current clinical guideline annotation or an FDA-approved drug label annotation, which must also be supported by at least one publication.

These combinations are reported as clinically helpful to guide dose adjustment or drug change depending on the metabolizing phenotype of the tested patient (poor metabolizer, intermediate metabolizer, normal metabolizer, rapid or ultra-rapid metabolizer) to increase efficacy or reduce toxicity. However, besides the recommendations provided by the international guidelines, CYP-based pharmacogenetic testing for antipsychotics is not widely implemented in clinical practice. Moreover, only a few studies on antipsychotic use in BD are available. In this regard, a recent meta-analysis [56] highlighted that most of the pharmacogenetic studies on antipsychotics performed so far have been carried out in patients with a diagnosis of schizophrenia. However, some studies have also included patients with BD, though the evidence remains too weak to draw firm conclusions. Nevertheless, this meta-analysis suggests that CYP-based pharmacogenetic testing may have clinical utility in guiding antipsychotic treatment, mainly using a multi-gene panel. This study also reveals high heterogeneity among studies and large variability in the frequency of actionable genetic variants across different ethnicities, stressing the need for more studies in this field.

In this regard, the ongoing PSY-PGx initiative [57] aims to perform a pharmacogenetic investigation in a cohort of 2500 patients diagnosed with various mental disorders who require antipsychotic treatment. This prospective, randomized, controlled study will evaluate the difference in recovery rates between the dosing-as-usual and the PGx-guided group of patients after dose adjustment according to international dosing recommendations for CYP2C19 and CYP2D6. When publicly available, the results from PSY-PGx will help provide valuable insights into the clinical utility and cost-effectiveness of PGx testing in antipsychotic treatment.

Moreover, the World Federation of Societies of Biological Psychiatry supports therapeutic drug monitoring (TDM) to personalize drug treatments, enhancing efficacy and minimizing side effects [58]. TDM represents a powerful tool that, based on clinical-chemical correlation data, enables tailored treatment for individual patients [59]. Inter-patient variability in pharmacokinetics, poor or non-response, and intolerability are primary patient-related reasons to apply TDM [58,60]. Indications for TDM in antipsychotic treatment extend well beyond common uses such as assessing adherence and managing polypharmacy. They include optimizing dosage to reach therapeutic drug concentrations, assessing drug levels in cases of poor response or side effects such as extrapyramidal symptoms (EPS) (e.g., amisulpride, haloperidol), determining the optimal maintenance dose when clinical improvement is accompanied by adverse effects, evaluating pharmacokinetic drug interactions and adjusting treatment based on clinical conditions such as pregnancy, older age, organ dysfunction, or genetic variations affecting drug metabolism [58].

Moreover, most of the evidence for antipsychotic switching strategies, frequency of exposure in BD, and, to a lesser extent [61], dosing in the management of acute mania [62] is still inferred from samples with schizophrenia, urging for additional ad-hoc pharmacogenomic-enriched trials specifically involving BD samples.

Increased gray matter density in the right inferior frontal gyrus, pro- and anti-inflammatory factors, lipid transport, metalloendopeptidase activity, cysteine protease inhibitor and growth factors may represent plausible biomarkers relevant to precision medicine in BD. Still, they warrant further validation [63], especially regarding predictors of antipsychotic response and tolerability.

Neuroimaging studies of antipsychotic response in BD are likewise outnumbered by their schizophrenia-sample counterpart [48,64]. However, such studies have provided valuable insights into the neural mechanisms underlying treatment response. For example, a previous study by [65] focused on the neurofunctional effects of quetiapine in patients with bipolar mania, revealing that after eight weeks of quetiapine monotherapy, patients with BD exhibited increased activation in the right orbitofrontal cortex, which was associated with clinical improvement in manic symptoms. This suggests that quetiapine may help restore prefrontal cortical activity, which is often disrupted in mania [65]. These findings underscore the potential of neuroimaging for monitoring treatment response and guiding therapeutic decisions.

Interestingly, a randomized, double-blind trial by [66] investigating the effects of short-term quetiapine and lithium therapy on the limbic system and emotion regulation circuitry in youth with acute manic or mixed episodes of BD type I found that quetiapine led to more rapid normalization of neural activation in regions such as the left amygdala, right putamen and right globus pallidus compared to lithium. Activation changes in the right putamen were correlated with reductions in manic symptoms, suggesting that quetiapine may induce more rapid functional brain changes in the limbic system and emotion regulation circuitry [66]. Interestingly, the same group demonstrated that pre-treatment morphometry and early morphometric changes (within the first week) independently predicted treatment response to quetiapine and lithium in youth with BD, with balanced accuracy exceeding 75% for both drugs [67]. These findings highlight the potential of functional and structural MRI data as candidate biomarkers to inform treatment stratification and guide biologically informed treatment decisions in BD. Further research is needed to refine predictive models and enhance precision in forecasting both antipsychotic efficacy and tolerability in individuals with BD.

## 4. Discussion

BD presents significant inter- and intra-individual clinical differences in symptoms, functioning, and response outcomes to antipsychotics across different phases and mood polarities [68,69,70] to such an extent that even the clinical pattern of antipsychotic management of BD may invariably often deviate from international treatment guidelines striving to cope with the most severe and complex clinical pictures [71].

In this scenario, precision antipsychotic management of BD is complex yet indispensable for improving the tolerability and safety of antipsychotic prescription and deprescription beyond the efficacy outcomes [72,73], moving toward proactive “P4” medicine (namely, “predictive”, “preventive”, “personalized”, and “participatory”) [74].

Precision antipsychotic management of BD does not aim to normalize individual differences. Instead, it focuses on customized treatment based on distinctive symptom patterns and the many clinical, psychopathological, genetic, neurobiological, and psychosocial variables we can currently discern and measure.

A “big” (actually, a “huge”) amount of “data” and variables contribute to different trajectories of antipsychotic treatment for BD. This has prompted the employment of high-end data science to analyze large amounts of information on biological and behavioral variables to detect latent classes and clusters of patients [75].

Treatment-relevant biomarkers are urgently needed for precise antipsychotic management of BD. However, the illness’s complex, polygenic, non-Mendelian nature and heterogeneous clinical phenotypes/stages [9] make this challenging [76].

So, what are the potential solutions to enhance the pursuit of precision antipsychotic medicine in BD?

The core of precision psychiatry is administering “the right drug for the right patient at the right time”. Such a high-bar pursuit must start with identifying biomarkers to improve the subtyping, classification, and reclassification of psychiatric disorders. These newly objectively identified subtypes would, in turn, lead to better antipsychotic management of BD. It is not an easy task, indeed [77].

Shared decision-making is also essential to enhance treatment adherence and overall outcomes of the antipsychotic treatment of BD.

Shared decision-making combines treatment algorithms and quality-ranked evidence with genetics and other data along with the preferences expressed by the person with living experience of BD (e.g., his/her propensity to tolerate specific potential treatment side effects to some extent, assuming an expected positive efficacy outcome is expected according to the quality-ranked evidence) and the prescribing clinician. For details, please see the “Preference tool for bipolar disorder” project (https://ebibd-database.org/preference/, accessed on 21 April 2025).

The combination of shared decisions with precision antipsychotic management of BD de facto represents an optimal balance between “precision” (focusing on “groups” of patients “accurately” clustered together based on the genetic and broad neurobiological makeup) and “personalized” medicine (an “individual-focused” approach focusing on specific issues such as the aforementioned therapeutic preferences and attitudes), two concepts often used interchangeably yet implying clinically relevant “nuances” against each other [78].

## 5. Conclusions

Overall, one may argue that the present work does not provide any novel insight or perspective compared to previous reports concerning the controversy surrounding precision medicine in BD [7,79,80]. It also implicitly aligns with the general considerations already made elsewhere about the potential economic and ethical issues raised by the precision antipsychotic management of BD [81,82,83,84].

While this apparently “inconclusive conclusion” may frustrate or disappoint some, the present succinct critical perspective nonetheless is “precise” in its goal to reinforce the need for specific research and clinical attention to the pharmacogenetics of antipsychotics in BD, which is still in its early phase.

In particular, while pharmacokinetics and pharmacodynamics considerations about the use of antipsychotics would not be affected by the underlying psychopathology per se, pharmacogenetics could, theoretically, vary, once again prompting for specific trials on BD samples to limit the inference about the safety and tolerability of antipsychotics in BD drawn from schizophrenia-sample studies since different SMIs may share substantial genetic underpinnings, yet they differ against each other.

This is especially important considering that these latter drugs, albeit crucial in the modern clinical management of BD, received far less attention compared to lithium or the controversial [85,86,87,88] antidepressant prescription in BD from a precision medicine approach.

## Figures and Tables

**Table 1:** Level of evidence for clinical utility of cytochrome genes in guiding treatment with antipsychotics according to the PharmGKB. (https://www.pharmgkb.org/).

Level	Gene	Drugs	Phenotype Categories
1A	CYP2D6	haloperidol	Metabolism/PK
1A	CYP2D6	zuclopenthixol	Metabolism/PK
1A	CYP2D6	aripiprazole	Metabolism/PK
1A	CYP2D6	risperidone	Metabolism/PK
1A	CYP3A4	quetiapine	Metabolism/PK

Legend: PK = pharmacokinetics.

## Abbreviations

The following abbreviations are used in this manuscript:

CYP	Cytochrome
BD	Bipolar Disorder
GWAS	Genome-wide association study
NMA	Network meta-analysis
TDM	Therapeutic drug monitoring
EPS	Extrapyramidal symptoms
RDoC	Research domain criteria
FDA	Food and Drug Administration
AI	Artificial intelligence
RCT	Randomized controlled trial
SMI	Severe mental illness
DSM	Diagnostic and Statical Manual for Mental Disorder
ICD	International Classification of Diseases
STEP-BD	Systematic treatment enhancement program for bipolar disorder
SNP	Single nucleotide polymorphism
